# Diurnal Regulation of Leaf Water Status in High- and Low-Mannitol Olive Cultivars

**DOI:** 10.3390/plants3020196

**Published:** 2014-03-25

**Authors:** Riccardo Lo Bianco, Giuseppe Avellone

**Affiliations:** 1 Dipartmento Scienze Agrarie e Forestali, Università degli Studi di Palermo, Viale delle Scienze 11, Palermo 90128, Italy; 2 Dipartimento Scienze e Tecnologie Biologiche Chimiche e Farmaceutiche, Università degli Studi di Palermo, Via Archirafi 32, Palermo 90123, Italy

**Keywords:** malate, polyol, relative water content, stomatal conductance, transpiration, vapor pressure deficit

## Abstract

The role of mannitol and malic acid in the regulation of diurnal leaf water relations was investigated in ‘Biancolilla’ (high-mannitol) and ‘Cerasuola’ (low-mannitol) olive trees. Photosynthetic photon flux density (PPFD), vapor pressure deficit (VPD), stomatal conductance (g_s_), transpiration rate (T), relative water content (RWC), mannitol and malic acid were measured in ‘Biancolilla’ and ‘Cerasuola’ leaves during a dry and hot day of summer in Sicily. In general, leaves of ‘Biancolilla’ trees exhibited greater mannitol content, higher g_s_ and T, but lower RWC than leaves of ‘Cerasuola’ trees. Differences in g_s_ and T between the two cultivars were evident mainly in mid to late morning. ‘Biancolilla’ leaves accumulated mannitol at midday and again late in the evening. Stomatal responses to VPD were RWC dependent, and limited somewhat T, only in ‘Biancolilla’. Mannitol was directly related to RWC, and may play an osmotic role, in ‘Biancolilla’ leaves, whereas ‘Cerasuola’ leaves remained well hydrated by just transpiring less and regardless of mannitol. A day-time accumulation and night-time utilization of mannitol in ‘Biancolilla’ leaves is proposed as an efficient mechanism to regulate water status and growth.

## 1. Introduction

Olive (*Olea europaea* L.) is native to and widespread in semi-arid regions where it commonly grows under high temperature and irradiation levels facing long periods of water deficit. Growth and yields of olive largely depend on the resistance to environmental stress [1,2].

During drought periods, olive is able to lower root osmotic potential and reduce leaf hydration, ending growth but keeping some photosynthesis and carbohydrate accumulation [3,4]. This has been mainly attributed to the accumulation of compatible solutes (osmotic adjustment). In particular, mannitol seems to contribute to osmotic adjustment under water deficit [3] and salt stress [5], and it increases in response to low temperatures *in vitro* [6], whereas sorbitol plays a similar role in apple [7], cherry [8], and peach [9]. In olive under severe water deficit, mannitol and glucose may be responsible for reductions of osmotic potential up to 0.32 MPa [4]. Also, in some species of the Oleaceae family, mannitol and malic acid contribute to the osmotic regulation of leaf water status in summer by accumulating around midday, when leaves are exposed to high solar radiation, temperatures and evaporative demand [10].

Sorbitol and mannitol are the most widely distributed sugar alcohols in the plant kingdom [11]. Mannitol is particularly abundant in species of Oleaceae, Apiaceae and Rubiaceae [11,12,13]. Once synthesized in mature leaves, mannitol may accumulate in source tissues or move to growing sinks [14].

In addition to playing an osmotic role, mannitol and other polyols are strong water-structure formers acting as effective stabilizing/protecting agents at both molecular and whole-cell level [15]. Polyols may also function as scavengers of reactive oxygen species and represent a non-enzymatic mechanism to protect cells from oxidative stress [16]. In transgenic plants exposed to salt or water stress, production of mannitol induces better survival and/or performance compared to wild types [17,18,19], and this does not seem to be attributable to osmotic protection by mannitol [18,20] but rather to a more specific radical scavenging mechanisms [21]. In olive, mannitol may function as a scavenger of oxygen radicals generated by paraquat applications or excess of light energy [22]. Finally, polyol production may enhance photosynthetic rates (similar to those of C4 species) as well as the efficiency of carbon and energy allocation [23,24].

Malic acid, on the other hand, is the most common organic acid in plant tissues [25,26], and it has been often considered a relevant component of osmotic adjustment during water deficit [27,28].

From previous investigations, we know that olive genotypes may significantly differ for their mannitol content in leaves and other organs [29]. In this study, the role of mannitol and malic acid in the regulation of diurnal leaf water relations was investigated using two olive cultivars with different leaf mannitol content.

## 2. Results and Discussion

Gas chromatography analyses confirmed that leaves of ‘Cerasuola’ olive had significantly less (only 62%) mannitol than leaves of ‘Biancolilla’ olive, whereas malic acid contents were similar in the two cultivars (Table 1). Leaves of ‘Biancolilla’ olive resulted also less hydrated than leaves of ‘Cerasuola’ olive, possibly due to greater water consumption by transpiration in the former cultivar (Table 1). Although a direct relationship between RWC and g_s_ has been documented in other species [30,31], and also observed in this study (discussed later, see Figure 5), the ability of ‘Biancolilla’ leaves to maintain high g_s_ and T under low RWC may be due to both greater osmotic adjustment and cell wall thickness (*i.e*., elasticity), which are known to confer dehydration tolerance to olive leaves [3,4].

**Table 1:** Average diurnal relative water content (RWC), stomatal conductance (g_s_), transpiration rate (T), mannitol content, and malic acid content in leaves of ‘Biancolilla’ and ‘Cerasuola’ olive trees on 18 August 2007 near Scillato, Sicily.

	Biancolilla	Cerasuola	*p* value ^1^
RWC (%)	78.3	87.1	<0.001
g_s _(mmol m^−2^ s^−1^)	110	68.0	<0.001
T (mmol m^−2^ s^−1^)	0.78	0.48	<0.001
Mannitol (mg g^−1^ DW)	770	477	<0.001
Malic acid (mg g^−1^ DW)	16.2	17.2	0.654

^1^ from analysis of variance.

**Figure 1:**
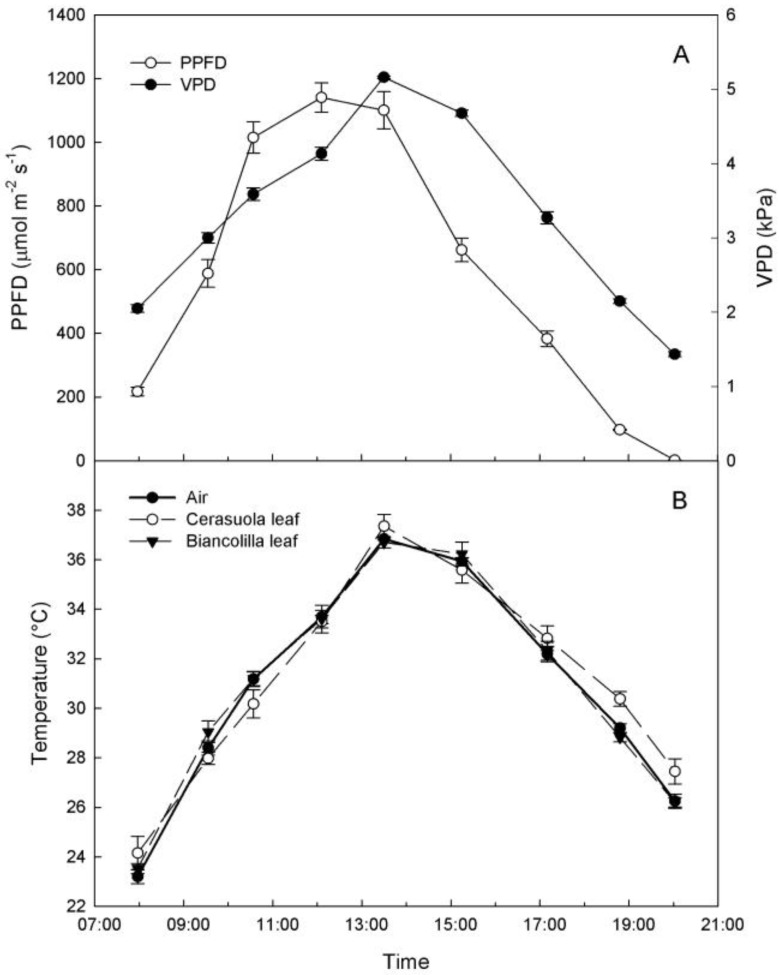
(**a**) Diurnal changes of photosynthetic photon flux density (PPFD) and vapor pressure deficit (VPD); (**b**) air temperature and temperatures of ‘Biancolilla’ and ‘Cerasuola’ olive leaves during a typical summer day in Sicily. Error bars represent standard errors of means.

As expected, ambient conditions on the sampling day were those of a typical hot and dry summer day in Sicily, with VPD reaching peak values of over 5 kPa around 13:30 HR, during the hours of highest light intensity (Figure 1A). Air and leaf temperatures followed trends similar to VPD, and only ‘Cerasuola’ leaves exhibited slightly higher temperatures than ‘Biancolilla’ leaves during evening hours (Figure 1B). 

**Figure 2:**
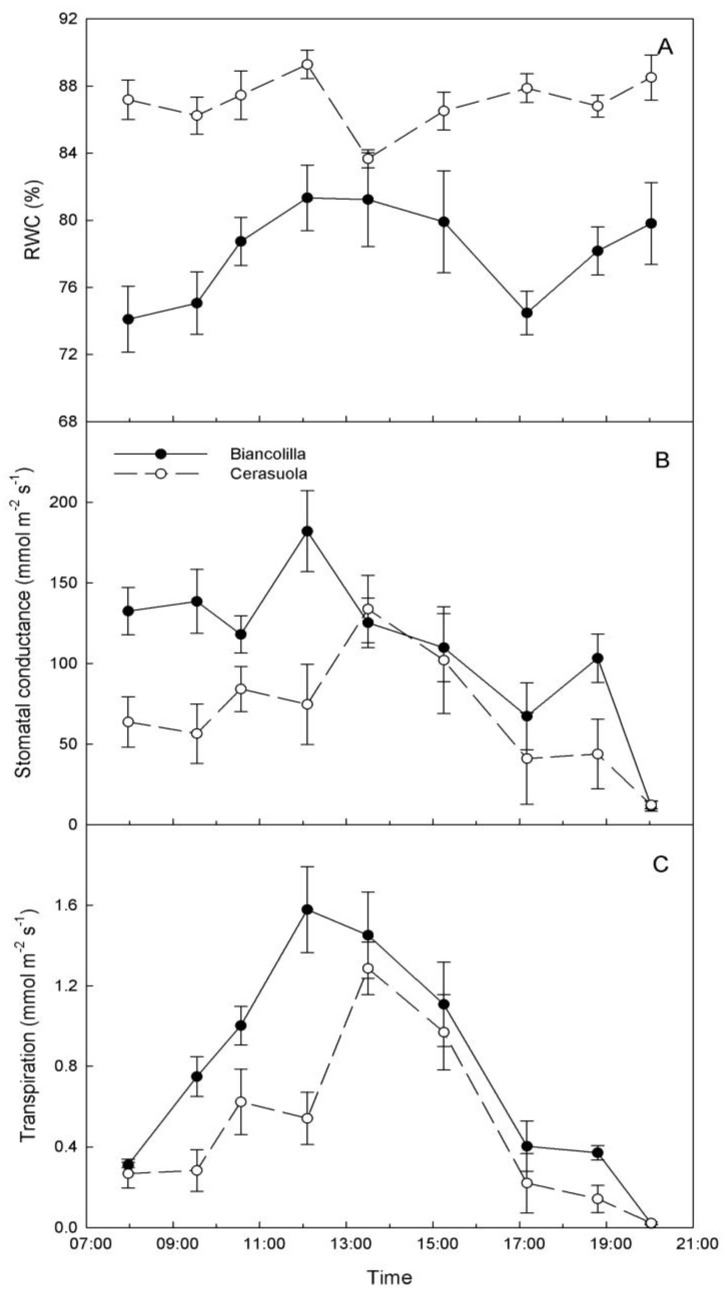
Diurnal changes of (**a**) relative water content (RWC); (**b**) stomatal conductance (g_s_); and (**c**) transpiration rate (T) in leaves of ‘Biancolilla’ and ‘Cerasuola’ olive trees during a typical summer day in Sicily. Error bars represent standard errors of means.

In ‘Cerasuola’ leaves, RWC levels did not exhibit significant diurnal changes, while RWC of ‘Biancolilla’ leaves exhibited low levels in the morning and late afternoon (Figure 2A). Also g_s_ exhibited different diurnal trends for the two cultivars (Figure 2B). In particular, g_s_ of ‘Biancolilla’ leaves was constantly higher than g_s_ of ‘Cerasuola’ leaves during morning hours and until noon; in the afternoon, g_s_ exhibited similar decreases in both cultivars, with the exception of 19:00 HR when g_s_ was again higher in ‘Biancolilla’ than in ‘Cerasuola’ leaves. In both cultivars, T followed the typical bell-shaped curve with an earlier peak in ‘Biancolilla’ (12:00 HR) than in ‘Cerasuola’ (13:30 HR) leaves, and major differences between the two cultivars during mid to late morning hours (Figure 2C). Within the general context of olive being indicated as a near-isohydric species [32,33], ‘Cerasuola’ seems to exhibit a more isohydric-like behavior than ‘Biancolilla’.

**Figure 3:**
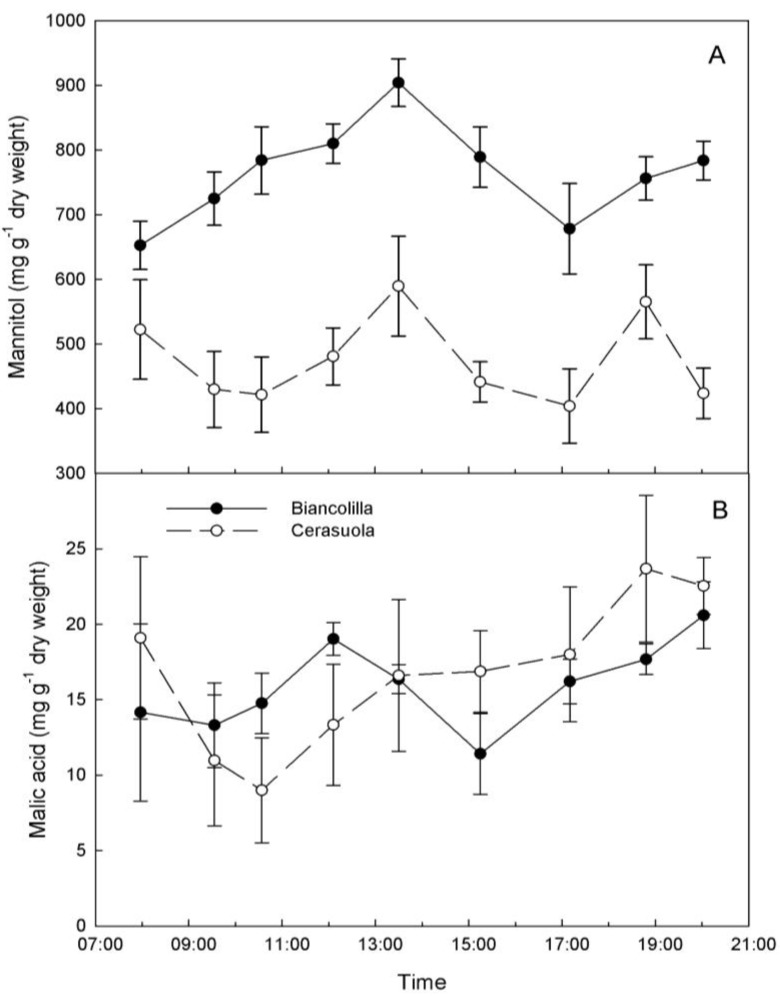
Diurnal changes of (**a**) mannitol and (**b**) malic acid in leaves of ‘Biancolilla’ and ‘Cerasuola’ olive trees during a typical summer day in Sicily. Error bars represent standard errors of means.

Mannitol content varied significantly during day hours reaching a peak around 13:30 HR in both cultivars (Figure 3A). Mannitol accumulated more in ‘Biancolilla’ than in ‘Cerasuola’ leaves during morning hours, decreased in the afternoon, and tended to resume accumulation in the evening only in ‘Biancolilla’ leaves. Morning accumulation of mannitol in ‘Biancolilla’ leaves may be the result of concomitant photosynthetic production (open stomates) and reduced translocation to sinks due to unfavorable hydration levels and low sink strength for mannitol. Similarly, accumulation of sorbitol in leaves of drought-stressed peach seedlings is caused by a decrease in sorbitol oxidation rates at the sink site and consequent reduction of its translocation [9].

On the other hand, malic acid content varied greatly across leaf samples, and this probably masked any significant change (*p* = 0.087) during day hours (Figure 3B). Formation of calcium chelates during sample freezing and thawing may also have affected malic acid quantification as suggested by Marigo and Peltier [27], although levels of malic acid observed in this study were three-folds higher then those previously reported in olive leaves [34]. In addition to the lack of a significant diurnal trend, malic acid levels were 30 to 50 times lower than mannitol levels, posing some doubts on the real contribution of malic acid to any possible osmotic adjustment in olive.

**Figure 4:**
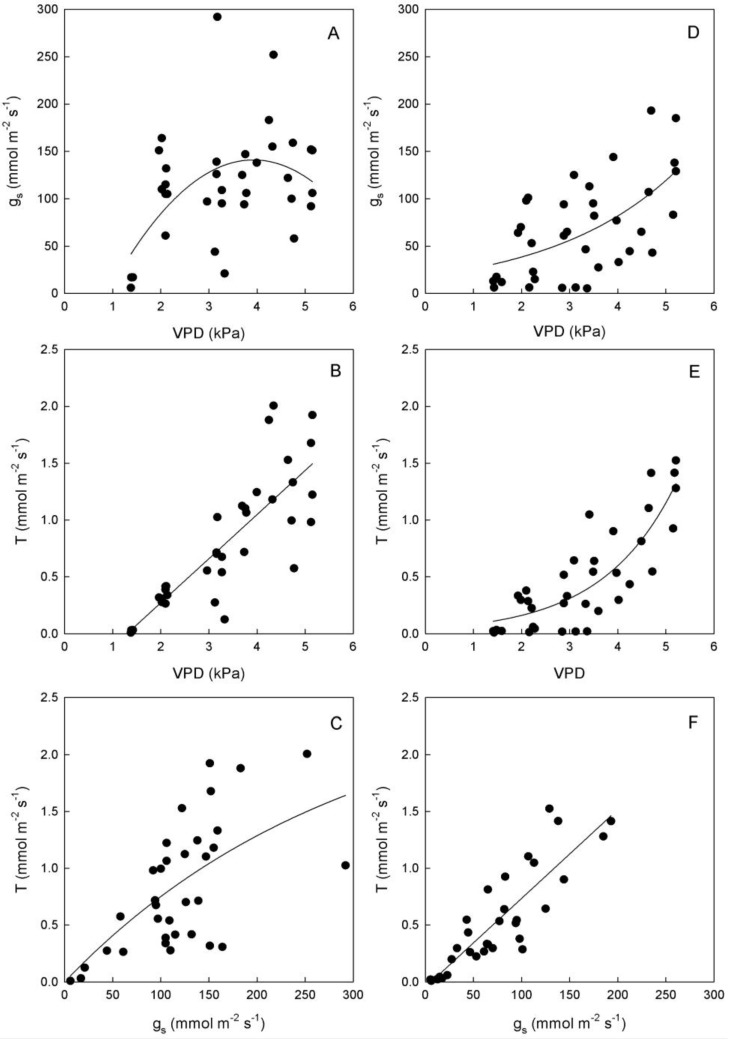
Relationships between vapor pressure deficit (VPD) and stomatal conductance (g_s_) or transpiration rate (T), and between g_s_ and T in leaves of (**A**–**C**) ‘Biancolilla’ and (**D**–**F**) ‘Cerasuola’ olive trees during a typical summer day in Sicily. In ‘Biancolilla’, T = Ln (0.83 + 0.013 g_s_); R^2^ = 0.486, *p* < 0.001; T = −0.50 + 0.39 VPD; R^2^ = 0.694, *p* < 0.001; g_s_ = −95 + 120 VPD − 15.3 VPD^2^; R^2^ = 0.265, *p* = 0.006. In ‘Cerasuola’, T = −0.05 + 0.008 g_s_; R^2^ = 0.782, *p* < 0.001; T = 0.04 × 1.93^VPD^; R^2^ = 0.706, *p* < 0.001; g_s_ = exp(2.90 + 0.38VPD); R^2^ = 0.353, *p* < 0.001. Models tested by regression analysis and best equation chosen according to Schwarz’s Bayesian Information Criterion.

Stomates tended to open as VPD increased in both cultivars, but with different trends (Figure 4). In particular, g_s_ of ‘Biancolilla’ leaves increased until VPD reached values of about 4 kPa, after which g_s_ started decreasing (Figure 4A); on the other hand, g_s_ of ‘Cerasuola’ leaves increased exponentially with VPD (Figure 4D) giving no sign of stomatal control on water loss. Also, T responded directly to VPD, according to a linear trend in ‘Biancolilla’ (Figure 4B) and to an exponential trend in ‘Cerasuola’ (Figure 4E). Finally, T was related to g_s_ according to a log-linear model in ‘Biancolilla’ (Figure 4C), whereas it responded linearly to g_s_ in ‘Cerasuola’ (Figure 4F). It is evident that, in the observed ranges, VPD was the main driving force for T, with g_s_ somewhat limiting T in ‘Biancolilla’ more than in ‘Cerasuola’. A direct association between VPD and T, but also g_s_ in absence of major dehydration, is expected during daily cycles. Low VPD levels were, in fact, associated to relatively low temperatures and light during early morning and late evening, and light and leaf temperature become the main factors driving T and g_s_. The observed differences in the stomatal behavior of the two cultivars may be at least in part due to the differences in leaf hydration level.

**Figure 5:**
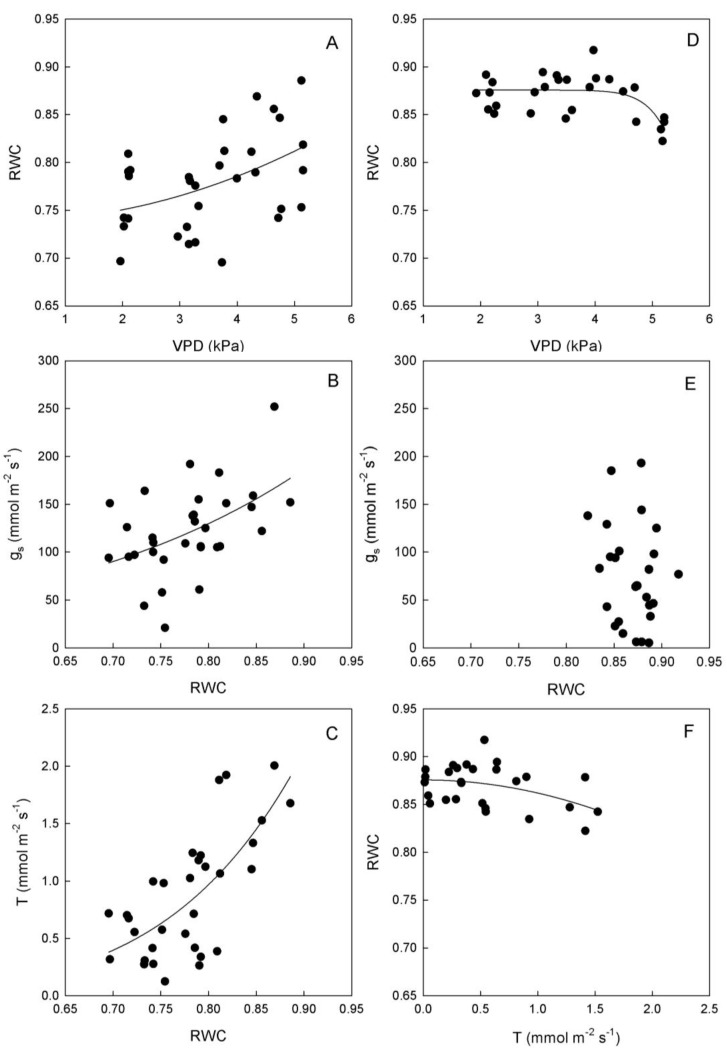
Relationships between relative water content (RWC) and vapor pressure deficit (VPD), stomatal conductance (g_s_), or transpiration rate (T) in leaves of (**A**–**C**) ‘Biancolilla’ and (**D**–**F**) ‘Cerasuola’ olive trees during a typical summer day in Sicily. In ‘Biancolilla’, g_s_ = exp(1.95 + 3.64 RWC); R^2^ = 0.234, *p* = 0.005; T = 4.29 RWC^6.68^; R^2^ = 0.500, *p* < 0.001. In ‘Cerasuola’, RWC = 0.88 + 0.0007 VPD^15^; R^2^ = 0.394, *p* = 0.003; T = 0.88 − 0.014 RWC^2^; R^2^ = 0.181, *p* = 0.027. Models tested by regression analysis and best equation chosen according to Schwarz’s Bayesian Information Criterion.

In ‘Biancolilla’, an unexpected increase in RWC was observed in response to VPD increases (Figure 5A); whereas in ‘Cerasuola’, RWC remained constant until a critical VPD level (around 4 kPa) beyond which RWC started decreasing (Figure 5D). T and g_s_ responses to RWC were also very different in the two cultivars. Specifically, RWC increases seem to determine exponential increases of both g_s_ and T in ‘Biancolilla’ (Figure 5 B,C). On the other hand, there was no relationship between RWC and g_s_ in ‘Cerasuola’ (Figure 5E), and rather T losses seem to be responsible for some leaf dehydration (Figure 5F). In this case, ‘Biancolilla’ leaves exhibited a passive feedback mechanism according to which RWC affects stomatal responses to VPD; conversely, ‘Cerasuola’ leaves seem to follow more of a feed forward mechanism where chemical signaling could affect stomatal responses to VPD [35].

**Figure 6:**
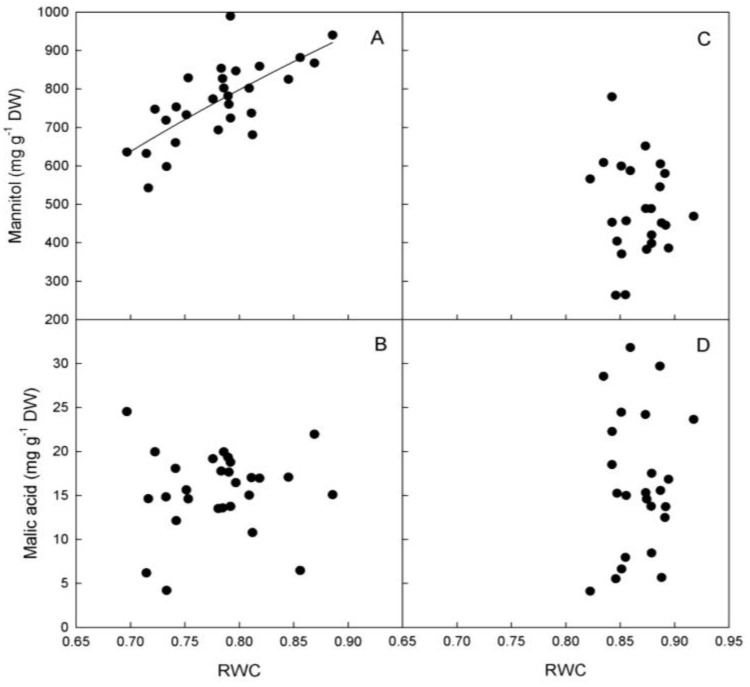
Relationships between mannitol, malic acid, and relative water content (RWC) in leaves of (**a**, **b**) ‘Biancolilla’ and (**c**, **d**) ‘Cerasuola’ olive trees during a typical summer day in Sicily. In ‘Biancolilla’, Mannitol = 1066 + 1203 Ln(RWC); R^2^ = 0.511, *p* < 0.001. Models tested by regression analysis and best equation chosen according to Schwarz’s Bayesian Information Criterion.

A positive association between RWC and mannitol was observed only in ‘Biancolilla’ leaves (Figure 6A), while no relationship was detected in ‘Cerasuola’ leaves (Figure 6C). No relationship was found between RWC and malic acid in either cultivar (Figure 6B,D). In ‘Biancolilla’, mannitol could play an active osmotic role and regulate leaf hydration level (increase turgor) and, in turn, g_s_ and T. Besides, it could also explain the unexpected increase of RWC as VPD increases. In particular, the accumulation of mannitol during evening hours seems very interesting and could be particularly important in terms of water and carbon balance during diurnal cycles. The amount of carbon in the form of mannitol accumulated in the evening could in fact be easily and efficiently translocated to sinks for growth and energy needs. At that point, the plant could maximize carbon, water, and energy use efficiency by producing and accumulating mannitol during daylight, when an osmotic function is needed, and translocating and using mannitol during dark hours when ambient conditions are more favorable for sink growth. It has been proposed indeed that mannitol, and polyols in general, can be a direct and efficient means for delivering carbon and energy to plant tissues and organs, as they yield NADH already in the first degradation step [23,24].

A similar mechanism was not evident in ‘Cerasuola’ leaves, where, in addition to stomatal control, some factor other than leaf mannitol (e.g., hydraulic conductivity and/or the ability to extract water from the soil) must be responsible for maintaining steadily high hydration levels during day hours.

## 3. Experimental

For the experiment, eight adult olive trees were selected, four of the cultivar Biancolilla and four of the cultivar Cerasuola. The two cultivars were chosen based on their different leaf mannitol content reported in a previous study [29]. Trees were all of the same age (20 years old), grown in the same grove (near Scillato, Sicily, 37°50'14''N, 13°56'47''E and 400 m a.s.l.), and treated with the same cultural cares and no irrigation.

All measurements and samples were collected on 18 August 2007, during a typical hot and dry summer day and after a period of over a month of no rain and maximum air temperatures above 30 °C. Hourly air temperature, relative humidity and solar radiation were acquired with a local meteorological station and used to calculate vapor pressure deficit (VPD).

Stomatal conductance (g_s_) was measured with an AP4 Delta-T porometer (Delta-T Devices, Cambridge, UK) at regular intervals from 8:00 to 20:00 HR (for a total of nine measurements per day) on one sun-exposed, mature, but non-senescent, leaf per tree. Transpiration (T) was estimated by converting VPD into vapor concentration deficit (mol m^−3^) and dividing by leaf resistance (m s^−1^). After each g_s_ measurement, the same leaf was wrapped in parafilm and aluminum foil, detached, stored in an ice-bag, and finally transported to the laboratory for determination of fresh weight (FW) with a four-digit precision scale. Fresh leaves were then rehydrated by immersing their petiole in distilled water for 24 h in the dark and at 8 °C, and weighed again for determination of turgid weight (TW). Finally, leaves were dried in a ventilated oven at 60 °C until constant weight and their dry weight (DW) was recorded. Relative water content (RWC) of each leaf was calculated as (FW − DW)/(TW − DW).

Leaves from the same shoots and opposite to the ones used for g_s_ and RWC measurements were sampled, transferred to the laboratory and stored at −40 °C for subsequent determination of mannitol and malic acid. Extraction was carried out using about 1 g of leaf blade, cut in small pieces, transferred into 1.5-mL eppendorf tubes and finely ground with a V-shaped pestle in presence of liquid nitrogen. Ground dry tissues were weighed and extracted with 1 mL of 80% (v:v) methanol solution containing 2.2 mg of phenyl-β-glucopiranoside as an internal standard. The homogenate was vortexed for 1 min and centrifuged for 5 min at 3000 g. The supernatant was stored at −40 °C for subsequent quantification of mannitol and malic acid with a Shimadzu 2010 gas chromatograph (GC) mounting an Equity 5 fast column (Sigma Aldrich, St. Louis, MO, USA). Sample preparation and GC method were those described in Lo Bianco *et al*. [29]. Briefly, 50-µL of extract was evaporated to dryness into 2-mL GC vials. Samples were derivatized by adding 75 µL of *N,O*-bis(trimethylsilyl)trifluoroacetamide and 15 µL of piridine, sonicating for 15 min and heating at 75 °C for 120 min. Calibration curves were constructed with separate standards for mannitol and malic acid using reagents from Sigma Aldrich.

Data were analyzed using GLM procedures of Systat software (Systat Software Inc., Richmond, CA, USA) with cultivar and time as main factors and cultivar x time as the sole interaction in the ANOVA model. Associations between response variables and for each cultivar were described by fitting linear or non-linear models with SigmaPlot (Systat Software Inc., Richmond, CA, USA). Schwarz’s Bayesian Information Criterion was used to compare regression models and choose best equation.

## 4. Conclusions

This study highlights two different strategies adopted by the two olive cultivars in order to cope with diurnal changes in atmospheric water demand. Specifically, ‘Cerasuola’ olive showed a sort of steady, conservative behavior as it tried to maintain favorable hydration levels by consuming less water and doing less in general. Conversely, ‘Biancolilla’ olive showed a more cyclic, definitely less conservative behavior as it kept stomates open causing some dehydration (day time), but allowing for production and accumulation of mannitol to possibly resume growth as soon as conditions become favorable (night time).
